# Bis(1,10-phenanthroline-κ^2^ *N*,*N*′)(sulfato-κ^2^ *O*,*O*′)cobalt(II) propane-1,2-diol monosolvate

**DOI:** 10.1107/S1600536812049616

**Published:** 2012-12-08

**Authors:** Kai-Long Zhong

**Affiliations:** a Department of Applied Chemistry, Nanjing College of Chemical Technology, Nanjing 210048, People’s Republic of China

## Abstract

In the title compound, [Co(SO_4_)(C_12_H_8_N_2_)_2_]·C_3_H_8_O_2_, the Co^II^ atom (site symmetry 2) has a distorted octa­hedral coordination composed of four N atoms from two chelating 1,10-phenanthroline ligands and two O atoms from an *O*,*O*′-bidentate sulfate ligand, in which the S atom has site symmetry 2. The dihedral angle between the two chelating N_2_C_2_ groups is 84.46 (15)°. The complex and solvent mol­ecules are connected through O—H⋯O hydrogen bonds. The solvent mol­ecule is equally disordered over two positions and is also located on a twofold axis.

## Related literature

The title complex has been reported with other solvant mol­ecules. In the case of ethane-1,2-diol, see: Zhong *et al.* (2006 ▶); for propane-1,3-diol, see: Zhong (2010 ▶); for butane-2,3-diol, see: Wang & Zhong (2011 ▶). For crystal engineering aspects of coordination framework structures, see: Batten & Robson (1998 ▶); Robin & Fromm (2006 ▶).
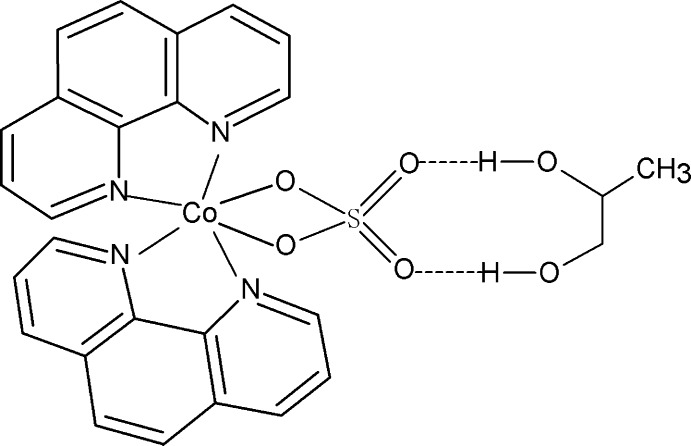


## Experimental

### 

#### Crystal data

[Co(SO_4_)(C_12_H_8_N_2_)_2_]·C_3_H_8_O_2_
*M* *_r_* = 591.49
Monoclinic, 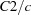

*a* = 18.117 (4) Å
*b* = 12.987 (3) Å
*c* = 12.881 (3) Å
β = 121.46 (3)°
*V* = 2585.2 (13) Å^3^
*Z* = 4
Mo *K*α radiation
μ = 0.80 mm^−1^
*T* = 223 K
0.35 × 0.34 × 0.25 mm

#### Data collection

Rigaku Mercury CCD diffractometer
Absorption correction: multi-scan (*REQAB*; Jacobson, 1998 ▶) *T*_min_ = 0.373, *T*_max_ = 1.000
11477 measured reflections
2284 independent reflections
1465 reflections with *I* > 2/s(*I*)
*R*_int_ = 0.100

#### Refinement

*R*[*F* ^2^ > 2σ(*F* ^2^)] = 0.052
*wR*(*F* ^2^) = 0.129
*S* = 0.95
2284 reflections
193 parameters
38 restraints
H-atom parameters constrained
Δρ_max_ = 0.37 e Å^−3^
Δρ_min_ = −0.39 e Å^−3^

### 

Data collection: *CrystalClear* (Rigaku, 2007 ▶); cell refinement: *CrystalClear*; data reduction: *CrystalClear*; program(s) used to solve structure: *SHELXS97* (Sheldrick, 2008 ▶); program(s) used to refine structure: *SHELXL97* (Sheldrick, 2008 ▶); molecular graphics: *XP* in *SHELXTL* (Sheldrick, 2008 ▶); software used to prepare material for publication: *SHELXTL*.

## Supplementary Material

Crystal structure: contains datablock(s) global, I. DOI: 10.1107/S1600536812049616/vn2062sup1.cif

Structure factors: contains datablock(s) I. DOI: 10.1107/S1600536812049616/vn2062Isup2.hkl

Additional supplementary materials:  crystallographic information; 3D view; checkCIF report

## Figures and Tables

**Table 1:** Selected bond lengths (Å)

Co1—O1	2.124 (3)
Co1—N1	2.123 (3)
Co1—N2	2.145 (4)

**Table 2:** Hydrogen-bond geometry (Å, °)

*D*—H⋯*A*	*D*—H	H⋯*A*	*D*⋯*A*	*D*—H⋯*A*
O3—H3⋯O2	0.82	1.95	2.698 (9)	150
O3′—H3′⋯O2	0.82	2.01	2.730 (10)	146

**Fig. 1.**
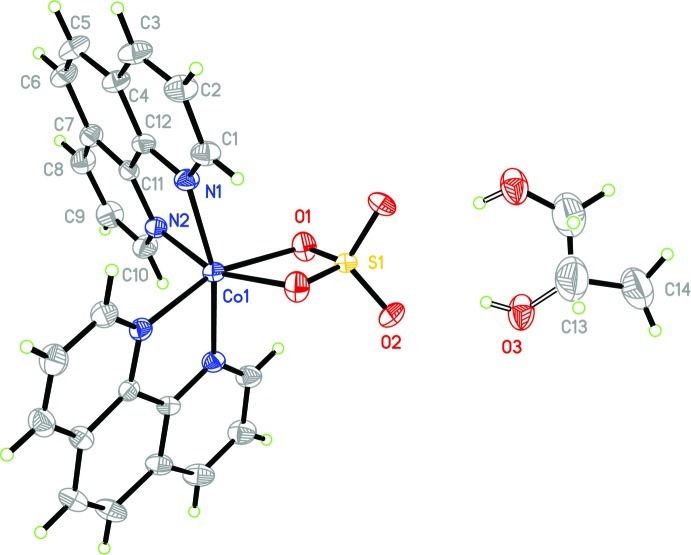
The molecular structure showing the atom-numbering scheme and with displacement ellipsoids drawn at the 30% probability level. Hydrogen bonds O—H···O are shown as dashed lines.

## supplementary crystallographic information

### Comment

In the past few decades, the supramolecular assembly and crystal engineering of metal-organic coordination frameworks have attracted much attention because of their potential applications in the areas of material chemistry (Batten & Robson, 1998; Robin & Fromm, 2006). Recently, we have unexpectedly obtained some cobalt-phen complexes with interesting four-membered chelating rings during attempts to synthesize mixed-ligand coordination polymers with phen as auxiliary ligand *via* a alcohol-solvothermal reaction, *e.g.* [CoSO_4_(C_12_H_8_N_2_)_2_]. C_2_H_6_O_2_ (Zhong *et al.*, 2006), (II), [CoSO_4_(C_12_H_8_N_2_)_2_].HOCH_2_ CH_2_CH_2_OH (Zhong, 2010), (III) and [CoSO_4_(C_12_H_8_N_2_)_2_]. C_4_H_10_O_2_ (Wang & Zhong, 2011), (IV). The crystal structure of the title compound [CuSO_4_(C_12_H_8_N_2_)_2_].C_3_H_8_O_2_, (I) has hitherto not been reported.

Single-crystal X-ray diffraction revealed that the asymmetric unit of (I) contains one neutral monomeric complex [CuSO_4_(C_12_H_8_N_2_)_2_] and one solvent propane-1,2-diol molecule, which are connected by an intermolecular O—H···O hydrogen bond with the uncoordinated O atoms of the sulfate group (Fig. 1 & Table 2). In the complex, a twofold rotation axis (symmetry code: 0, *y*, 1/4) passes through the Co^II^ atom and the S atom. The Co^II^ atom has a distorted CoN_4_O_2_ octahedral geometry, with four N atoms from two chelating phenanthroline ligands and two O atoms from an O,*O*'-bidentate sulfate anion (Fig. 1). The Co—O bond distance [2.124 (3) Å], the Co—N bond distance [2.123 (3) Å], the N—Co—N bite angles [77.50 (13)°] and O—Co—O bite angle [66.91 (16)°] are within normal ranges and are comparable to the closely related structure (II) - (IV). The two chelating N_2_C_2_ groups are oriented at 84.46 (15)°, which is much larger than reported in (II), (III) and (IV) [70.16 (6)°, 80.06 (8)° and 83.48 (1)°, respectively]. The solvent molecule is disordered over two sets of sites with occupancies of 0.50 and 0.50.

### Experimental

0.2 mmol phen, 0.1 mmol melamine, 0.1 mmol CoSO_4_.7H_2_O, 2.0 ml propane-1,2-diol and 1.0 ml water were mixed and placed in a thick Pyrex tube, which was sealed and heated to 453 K for 96 h, whereupon red block-shaped crystals of the title compound were obtained.

### Refinement

All non-hydrogen atoms were refined anisotropically. The H atoms of phen were positioned geometrically and allowed to ride on their parent atoms, with C—H = 0.93 Å and *U*_iso_(H) = 1.2*U*_eq_(C). The H atoms of propane-1,2-diol were placed in geometrically idealized positions and refined as riding atoms, with C—H(CH_3_) = 0.96 Å, C—H(CH_2_) = 0.97 Å, C—H(CH) = 0.98 Å and O—H = 0.82 Å; *U*_iso_(H) = 1.2*U*_eq_(C) and 1.5*U*_eq_(O). The propane-1,2-diol molecule was found to be disordered over two positions with site occupancy factors of 0.50:0.50. The site occupancy factors were not refined. In order to keep a reasonable geometry distance restraints were used, apart from atomic displacement parameter restraints (ISOR and EADP).

### Crystal data

**Table:**

[Co(SO_4_)(C_12_H_8_N_2_)_2_]·C_3_H_8_O_2_	*F*(000) = 1220
*M**_r_* = 591.49	*D*_x_ = 1.520 Mg m^−^^3^
Monoclinic, *C*2/*c*	Mo *K*α radiation, λ = 0.71073 Å
Hall symbol: -C 2yc	Cell parameters from 5104 reflections
*a* = 18.117 (4) Å	θ = 3.1–25.4°
*b* = 12.987 (3) Å	µ = 0.80 mm^−^^1^
*c* = 12.881 (3) Å	*T* = 223 K
β = 121.46 (3)°	Block, red
*V* = 2585.2 (13) Å^3^	0.35 × 0.34 × 0.25 mm
*Z* = 4	

### Data collection

**Table:**

Rigaku Mercury CCD diffractometer	2284 independent reflections
Radiation source: fine-focus sealed tube	1465 reflections with *I* > 2/s(*I*)
Graphite Monochromator monochromator	*R*_int_ = 0.100
Detector resolution: 28.5714 pixels mm^-1^	θ_max_ = 25.0°, θ_min_ = 3.1°
ω scans	*h* = −20→21
Absorption correction: multi-scan (*REQAB*; Jacobson, 1998)	*k* = −14→15
*T*_min_ = 0.373, *T*_max_ = 1.000	*l* = −15→15
11477 measured reflections	

### Refinement

**Table:**

Refinement on *F*^2^	Secondary atom site location: difference Fourier map
Least-squares matrix: full	Hydrogen site location: inferred from neighbouring sites
*R*[*F*^2^ > 2σ(*F*^2^)] = 0.052	H-atom parameters constrained
*wR*(*F*^2^) = 0.129	*w* = 1/[σ^2^(*F*_o_^2^) + (0.0385*P*)^2^] where *P* = (*F*_o_^2^ + 2*F*_c_^2^)/3
*S* = 0.95	(Δ/σ)_max_ = 0.001
2284 reflections	Δρ_max_ = 0.37 e Å^−^^3^
193 parameters	Δρ_min_ = −0.39 e Å^−^^3^
38 restraints	Extinction correction: *SHELXL97* (Sheldrick, 2008), Fc^*^=kFc[1+0.001xFc^2^λ^3^/sin(2θ)]^-1/4^
Primary atom site location: structure-invariant direct methods	Extinction coefficient: 0.0060 (5)

### Special details

**Table:**

Geometry. All e.s.d.'s (except the e.s.d. in the dihedral angle between two l.s. planes) are estimated using the full covariance matrix. The cell e.s.d.'s are taken into account individually in the estimation of e.s.d.'s in distances, angles and torsion angles; correlations between e.s.d.'s in cell parameters are only used when they are defined by crystal symmetry. An approximate (isotropic) treatment of cell e.s.d.'s is used for estimating e.s.d.'s involving l.s. planes.
Refinement. Refinement of *F*^2^ against ALL reflections. The weighted *R*-factor *wR* and goodness of fit *S* are based on *F*^2^, conventional *R*-factors *R* are based on *F*, with *F* set to zero for negative *F*^2^. The threshold expression of *F*^2^ > σ(*F*^2^) is used only for calculating *R*-factors(gt) *etc*. and is not relevant to the choice of reflections for refinement. *R*-factors based on *F*^2^ are statistically about twice as large as those based on *F*, and *R*- factors based on ALL data will be even larger.

### Fractional atomic coordinates and isotropic or equivalent isotropic displacement parameters (Å^2^)

**Table:**

	*x*	*y*	*z*	*U*_iso_*/*U*_eq_	Occ. (<1)
Co1	0.0000	0.32137 (6)	0.2500	0.0348 (3)	
S1	0.0000	0.52899 (11)	0.2500	0.0344 (4)	
O1	0.0533 (2)	0.4578 (2)	0.3537 (3)	0.0495 (9)	
O2	−0.0552 (2)	0.5929 (2)	0.2742 (3)	0.0604 (10)	
O3	−0.0492 (7)	0.7963 (6)	0.3204 (8)	0.071 (3)	0.50
H3	−0.0428	0.7424	0.2931	0.106*	0.50
O3'	−0.0869 (6)	0.7996 (6)	0.2438 (10)	0.097 (4)	0.50
H3'	−0.0613	0.7472	0.2804	0.145*	0.50
N1	0.0830 (2)	0.3000 (3)	0.1814 (3)	0.0379 (9)	
N2	0.0959 (2)	0.2178 (2)	0.3803 (3)	0.0363 (9)	
C1	0.0774 (3)	0.3442 (4)	0.0841 (4)	0.0459 (12)	
H1A	0.0340	0.3924	0.0406	0.055*	
C2	0.1339 (3)	0.3208 (4)	0.0454 (5)	0.0537 (14)	
H2A	0.1284	0.3535	−0.0225	0.064*	
C3	0.1977 (3)	0.2495 (4)	0.1074 (5)	0.0555 (14)	
H3A	0.2354	0.2331	0.0814	0.067*	
C4	0.2061 (3)	0.2012 (3)	0.2104 (4)	0.0419 (12)	
C5	0.2732 (3)	0.1286 (4)	0.2841 (5)	0.0538 (14)	
H5A	0.3117	0.1082	0.2608	0.065*	
C6	0.2814 (3)	0.0902 (4)	0.3853 (5)	0.0493 (13)	
H6A	0.3258	0.0440	0.4320	0.059*	
C7	0.2225 (3)	0.1189 (3)	0.4240 (4)	0.0402 (11)	
C8	0.2298 (3)	0.0829 (3)	0.5312 (4)	0.0506 (13)	
H8A	0.2738	0.0375	0.5816	0.061*	
C9	0.1723 (3)	0.1150 (4)	0.5605 (4)	0.0546 (14)	
H9A	0.1767	0.0924	0.6320	0.065*	
C10	0.1062 (3)	0.1819 (3)	0.4833 (4)	0.0468 (13)	
H10A	0.0671	0.2026	0.5053	0.056*	
C11	0.1546 (3)	0.1866 (3)	0.3517 (4)	0.0320 (10)	
C12	0.1473 (3)	0.2300 (3)	0.2447 (4)	0.0341 (11)	
C13	−0.0288 (6)	0.8772 (5)	0.2729 (9)	0.139 (3)	0.50
H13	−0.0837	0.8822	0.1948	0.167*	0.50
C13'	−0.0288 (6)	0.8772 (5)	0.2729 (9)	0.139 (3)	0.50
H13A	0.0069	0.8799	0.3610	0.167*	0.50
H13B	−0.0613	0.9411	0.2467	0.167*	0.50
C14	−0.0305 (12)	0.9804 (8)	0.3222 (18)	0.168 (9)	0.50
H14A	0.0001	1.0290	0.3021	0.252*	0.50
H14B	−0.0032	0.9762	0.4091	0.252*	0.50
H14C	−0.0894	1.0025	0.2872	0.252*	0.50

### Atomic displacement parameters (Å^2^)

**Table:**

	*U*^11^	*U*^22^	*U*^33^	*U*^12^	*U*^13^	*U*^23^
Co1	0.0323 (5)	0.0367 (5)	0.0407 (6)	0.000	0.0228 (5)	0.000
S1	0.0282 (9)	0.0352 (9)	0.0424 (10)	0.000	0.0203 (8)	0.000
O1	0.048 (2)	0.0428 (18)	0.038 (2)	−0.0012 (15)	0.0091 (17)	0.0020 (15)
O2	0.060 (2)	0.049 (2)	0.095 (3)	0.0103 (17)	0.056 (2)	−0.0082 (19)
O3	0.111 (7)	0.050 (5)	0.077 (6)	0.005 (4)	0.068 (5)	−0.001 (4)
O3'	0.110 (7)	0.067 (6)	0.126 (8)	0.026 (5)	0.070 (6)	0.023 (6)
N1	0.038 (2)	0.042 (2)	0.041 (2)	0.0010 (17)	0.026 (2)	0.0036 (18)
N2	0.041 (2)	0.036 (2)	0.042 (2)	−0.0010 (17)	0.029 (2)	0.0011 (17)
C1	0.041 (3)	0.056 (3)	0.044 (3)	0.003 (2)	0.024 (3)	0.007 (2)
C2	0.054 (3)	0.070 (4)	0.046 (3)	0.003 (3)	0.033 (3)	0.008 (3)
C3	0.050 (3)	0.080 (4)	0.052 (3)	0.004 (3)	0.038 (3)	−0.003 (3)
C4	0.036 (3)	0.057 (3)	0.036 (3)	0.004 (2)	0.021 (2)	−0.002 (2)
C5	0.044 (3)	0.073 (4)	0.050 (3)	0.018 (3)	0.028 (3)	−0.001 (3)
C6	0.037 (3)	0.059 (3)	0.047 (3)	0.015 (2)	0.018 (3)	0.001 (3)
C7	0.036 (3)	0.042 (3)	0.038 (3)	0.005 (2)	0.017 (2)	0.002 (2)
C8	0.046 (3)	0.049 (3)	0.047 (3)	0.007 (2)	0.018 (3)	0.006 (2)
C9	0.063 (4)	0.059 (3)	0.046 (3)	0.005 (3)	0.031 (3)	0.015 (3)
C10	0.056 (3)	0.050 (3)	0.049 (3)	0.003 (2)	0.038 (3)	0.005 (2)
C11	0.033 (3)	0.031 (2)	0.034 (3)	−0.0038 (19)	0.019 (2)	−0.0020 (19)
C12	0.027 (3)	0.041 (3)	0.034 (3)	−0.001 (2)	0.015 (2)	−0.002 (2)
C13	0.195 (9)	0.076 (5)	0.215 (9)	0.002 (5)	0.155 (7)	−0.006 (6)
C13'	0.195 (9)	0.076 (5)	0.215 (9)	0.002 (5)	0.155 (7)	−0.006 (6)
C14	0.20 (2)	0.069 (10)	0.32 (3)	−0.029 (11)	0.19 (2)	−0.032 (14)

### Geometric parameters (Å, º)

**Table:**

Co1—O1	2.124 (3)	C3—C4	1.402 (6)
Co1—O1^i^	2.124 (3)	C3—H3A	0.9300
Co1—N1	2.123 (3)	C4—C12	1.400 (6)
Co1—N1^i^	2.123 (3)	C4—C5	1.437 (6)
Co1—N2^i^	2.145 (4)	C5—C6	1.330 (6)
Co1—N2	2.145 (4)	C5—H5A	0.9300
Co1—S1	2.6964 (18)	C6—C7	1.442 (6)
S1—O2^i^	1.453 (3)	C6—H6A	0.9300
S1—O2	1.453 (3)	C7—C11	1.399 (6)
S1—O1^i^	1.492 (3)	C7—C8	1.397 (6)
S1—O1	1.492 (3)	C8—C9	1.346 (6)
O3—C13	1.361 (4)	C8—H8A	0.9300
O3—H3	0.8200	C9—C10	1.391 (6)
O3'—H3'	0.8200	C9—H9A	0.9300
N1—C1	1.333 (5)	C10—H10A	0.9300
N1—C12	1.363 (5)	C11—C12	1.430 (6)
N2—C10	1.324 (5)	C13—C13^i^	1.443 (7)
N2—C11	1.356 (5)	C13—C14	1.489 (9)
C1—C2	1.386 (6)	C13—H13	0.9800
C1—H1A	0.9300	C14—H14A	0.9600
C2—C3	1.366 (6)	C14—H14B	0.9600
C2—H2A	0.9300	C14—H14C	0.9600
			
O1—Co1—O1^i^	66.91 (16)	C1—C2—H2A	120.2
O1—Co1—N1	100.51 (13)	C2—C3—C4	119.9 (4)
O1^i^—Co1—N1	92.02 (13)	C2—C3—H3A	120.1
O1—Co1—N1^i^	92.02 (13)	C4—C3—H3A	120.1
O1^i^—Co1—N1^i^	100.51 (13)	C12—C4—C3	117.0 (4)
N1—Co1—N1^i^	165.01 (18)	C12—C4—C5	119.2 (4)
O1—Co1—N2^i^	158.77 (12)	C3—C4—C5	123.8 (4)
O1^i^—Co1—N2^i^	96.57 (12)	C6—C5—C4	121.1 (4)
N1—Co1—N2^i^	93.03 (13)	C6—C5—H5A	119.5
N1^i^—Co1—N2^i^	77.50 (13)	C4—C5—H5A	119.5
O1—Co1—N2	96.57 (12)	C5—C6—C7	121.2 (4)
O1^i^—Co1—N2	158.77 (12)	C5—C6—H6A	119.4
N1—Co1—N2	77.50 (13)	C7—C6—H6A	119.4
N1^i^—Co1—N2	93.03 (13)	C11—C7—C8	117.7 (4)
N2^i^—Co1—N2	102.31 (18)	C11—C7—C6	119.0 (4)
O1—Co1—S1	33.45 (8)	C8—C7—C6	123.3 (4)
O1^i^—Co1—S1	33.45 (8)	C9—C8—C7	119.0 (4)
N1—Co1—S1	97.50 (9)	C9—C8—H8A	120.5
N1^i^—Co1—S1	97.50 (9)	C7—C8—H8A	120.5
N2^i^—Co1—S1	128.85 (9)	C8—C9—C10	119.7 (5)
N2—Co1—S1	128.85 (9)	C8—C9—H9A	120.1
O2^i^—S1—O2	110.3 (3)	C10—C9—H9A	120.1
O2^i^—S1—O1^i^	111.01 (19)	N2—C10—C9	123.8 (4)
O2—S1—O1^i^	110.46 (19)	N2—C10—H10A	118.1
O2^i^—S1—O1	110.46 (19)	C9—C10—H10A	118.1
O2—S1—O1	111.01 (19)	N2—C11—C7	123.3 (4)
O1^i^—S1—O1	103.4 (2)	N2—C11—C12	116.9 (4)
O2^i^—S1—Co1	124.83 (13)	C7—C11—C12	119.7 (4)
O2—S1—Co1	124.83 (13)	N1—C12—C4	122.9 (4)
O1^i^—S1—Co1	51.70 (12)	N1—C12—C11	117.4 (4)
O1—S1—Co1	51.70 (12)	C4—C12—C11	119.8 (4)
S1—O1—Co1	94.85 (16)	O3—C13—C13^i^	127.5 (5)
C13—O3—H3	109.5	O3—C13—C14	115.6 (8)
C1—N1—C12	118.1 (4)	C13^i^—C13—C14	110.8 (7)
C1—N1—Co1	127.8 (3)	O3—C13—H13	98.3
C12—N1—Co1	114.1 (3)	C13^i^—C13—H13	98.3
C10—N2—C11	116.5 (4)	C14—C13—H13	98.3
C10—N2—Co1	129.7 (3)	C13—C14—H14A	109.5
C11—N2—Co1	113.9 (3)	C13—C14—H14B	109.5
N1—C1—C2	122.4 (4)	H14A—C14—H14B	109.5
N1—C1—H1A	118.8	C13—C14—H14C	109.5
C2—C1—H1A	118.8	H14A—C14—H14C	109.5
C3—C2—C1	119.7 (5)	H14B—C14—H14C	109.5
C3—C2—H2A	120.2		
			
O1—Co1—S1—O2^i^	89.6 (2)	N2^i^—Co1—N2—C10	−94.6 (4)
O1^i^—Co1—S1—O2^i^	−90.4 (2)	S1—Co1—N2—C10	85.4 (4)
N1—Co1—S1—O2^i^	−8.1 (2)	O1—Co1—N2—C11	−103.3 (3)
N1^i^—Co1—S1—O2^i^	171.9 (2)	O1^i^—Co1—N2—C11	−65.9 (5)
N2^i^—Co1—S1—O2^i^	−108.2 (2)	N1—Co1—N2—C11	−4.0 (3)
N2—Co1—S1—O2^i^	71.8 (2)	N1^i^—Co1—N2—C11	164.3 (3)
O1—Co1—S1—O2	−90.4 (2)	N2^i^—Co1—N2—C11	86.4 (3)
O1^i^—Co1—S1—O2	89.6 (2)	S1—Co1—N2—C11	−93.6 (3)
N1—Co1—S1—O2	171.9 (2)	C12—N1—C1—C2	0.4 (7)
N1^i^—Co1—S1—O2	−8.1 (2)	Co1—N1—C1—C2	−177.4 (3)
N2^i^—Co1—S1—O2	71.8 (2)	N1—C1—C2—C3	0.5 (8)
N2—Co1—S1—O2	−108.2 (2)	C1—C2—C3—C4	−0.6 (8)
O1—Co1—S1—O1^i^	180.0	C2—C3—C4—C12	−0.3 (7)
N1—Co1—S1—O1^i^	82.27 (19)	C2—C3—C4—C5	−177.3 (5)
N1^i^—Co1—S1—O1^i^	−97.73 (19)	C12—C4—C5—C6	−1.3 (7)
N2^i^—Co1—S1—O1^i^	−17.8 (2)	C3—C4—C5—C6	175.6 (5)
N2—Co1—S1—O1^i^	162.2 (2)	C4—C5—C6—C7	0.8 (8)
O1^i^—Co1—S1—O1	180.0	C5—C6—C7—C11	1.5 (7)
N1—Co1—S1—O1	−97.73 (19)	C5—C6—C7—C8	−178.2 (5)
N1^i^—Co1—S1—O1	82.27 (19)	C11—C7—C8—C9	−0.4 (7)
N2^i^—Co1—S1—O1	162.2 (2)	C6—C7—C8—C9	179.2 (4)
N2—Co1—S1—O1	−17.8 (2)	C7—C8—C9—C10	0.8 (7)
O2^i^—S1—O1—Co1	−118.82 (17)	C11—N2—C10—C9	−0.6 (7)
O2—S1—O1—Co1	118.44 (18)	Co1—N2—C10—C9	−179.5 (3)
O1^i^—S1—O1—Co1	0.0	C8—C9—C10—N2	−0.4 (8)
O1^i^—Co1—O1—S1	0.0	C10—N2—C11—C7	1.0 (6)
N1—Co1—O1—S1	87.70 (17)	Co1—N2—C11—C7	−179.9 (3)
N1^i^—Co1—O1—S1	−100.56 (16)	C10—N2—C11—C12	−176.1 (4)
N2^i^—Co1—O1—S1	−41.1 (4)	Co1—N2—C11—C12	3.0 (5)
N2—Co1—O1—S1	166.15 (15)	C8—C7—C11—N2	−0.6 (7)
O1—Co1—N1—C1	−83.2 (4)	C6—C7—C11—N2	179.8 (4)
O1^i^—Co1—N1—C1	−16.3 (4)	C8—C7—C11—C12	176.5 (4)
N1^i^—Co1—N1—C1	130.6 (4)	C6—C7—C11—C12	−3.1 (6)
N2^i^—Co1—N1—C1	80.4 (4)	C1—N1—C12—C4	−1.4 (6)
N2—Co1—N1—C1	−177.6 (4)	Co1—N1—C12—C4	176.7 (3)
S1—Co1—N1—C1	−49.4 (4)	C1—N1—C12—C11	177.4 (4)
O1—Co1—N1—C12	99.0 (3)	Co1—N1—C12—C11	−4.5 (5)
O1^i^—Co1—N1—C12	165.9 (3)	C3—C4—C12—N1	1.3 (7)
N1^i^—Co1—N1—C12	−47.3 (3)	C5—C4—C12—N1	178.4 (4)
N2^i^—Co1—N1—C12	−97.5 (3)	C3—C4—C12—C11	−177.5 (4)
N2—Co1—N1—C12	4.5 (3)	C5—C4—C12—C11	−0.4 (6)
S1—Co1—N1—C12	132.7 (3)	N2—C11—C12—N1	1.0 (6)
O1—Co1—N2—C10	75.6 (4)	C7—C11—C12—N1	−176.3 (4)
O1^i^—Co1—N2—C10	113.1 (5)	N2—C11—C12—C4	179.9 (4)
N1—Co1—N2—C10	175.0 (4)	C7—C11—C12—C4	2.6 (6)
N1^i^—Co1—N2—C10	−16.8 (4)		

Symmetry code: (i) −*x*, *y*, −*z*+1/2.

### Hydrogen-bond geometry (Å, º)

**Table:**

*D*—H···*A*	*D*—H	H···*A*	*D*···*A*	*D*—H···*A*
O3—H3···O2	0.82	1.95	2.698 (9)	150
O3′—H3′···O2	0.82	2.01	2.730 (10)	146
